# Neuromodulation of the Pudendal Nerve Assisted by 3D Printed: A New Method of Neuromodulation for Lower Urinary Tract Dysfunction

**DOI:** 10.3389/fnins.2021.619672

**Published:** 2021-02-26

**Authors:** Yinjun Gu, Tingting Lv, Chen Jiang, Jianwei Lv

**Affiliations:** Department of Urology, Renji Hospital, Shanghai Jiao Tong University School of Medicine, Shanghai, China

**Keywords:** 3D printing, nerve stimulation, pudendal nerve, image fusion, sacral nerve

## Abstract

Electrical stimulation of peripheral nerves by implanted electrodes is an effective treatment for certain pelvic floor diseases. As well as intravesical electrical stimulation, this predominantly includes stimulation of the sacral nerve, tibial nerve, and pudendal nerve. The pudendal nerve is one of the main nerves that stimulate pelvic floor muscles, external urethral meatus, and the anal sphincter and pelvic organs, and it may have effects on frequent urination, urgency, dysuria, and perineal pain. It is difficult to locate because of its anatomical course, however, leading to difficulties fixing the electrode, which increases the difficulty of pudendal nerve electrical stimulation in clinical practice. In the current study 3D printed navigation was used to solve these problems. Combined with autopsy data and patient pelvic and nerve data, a personalized design was generated. Neural modulation of the pudendal nerve was achieved by implanting the lead with the guidance of 3D printed navigation. 3D printed navigation can maximize the phase II conversion rate, reduce the difficulty of surgery, shorten the operation time, reduce damage to additional organs and blood vessels, and increase the accuracy of electrode implantation, and it can be performed while the patient is awake. It is an accurate, reversible, efficient, and minimally invasive surgery.

## Introduction

Neuromodulation is an emerging medical field, and it has been shown to be an effective treatment for a variety of pelvic floor diseases and other diseases such as overactive bladder (Salima et al., 2017), interstitial cystitis (Han et al., 2018), and neurogenic bladder (Kessler et al., 2010). The neural regulation of pelvic floor diseases mainly utilizes sacral nerve regulation (Zhang et al., 2019). Sacral neuromodulation therapy was proposed by Schmidt et al. (1979) for the treatment options of lower urinary tract functional disorders. In 2012, China officially launched a large-scale promotion of SNM therapy. In the past 5 years, nearly 60 centers in China have carried out SNM clinical studies, and there are also several clinical articles published in China that report the efficacy and prognosis of SNM (Chen et al., 2014; Zhang et al., 2017). Researchers are also exploring other pelvic floor and peripheral nerves as new regulatory targets to achieve better efficacy and reduce associated complications, such as the pudendal nerve (Marinkovic et al., 2015) and the tibial nerve (Bhide et al., 2020). The pudendal nerve is one of the main nerves that stimulates pelvic floor muscles, external urethral meatus, the anal sphincter, and pelvic organs, and it is composed of nerve fibers emanating from S2 to S4 nerve roots. It is a mixed nerve containing somatic and autonomic nerve fibers. Because the pudendal nerve has no bony localization mark, so its surgical localization and puncture are problematic.

Reitz et al. (2007) described a new method of pudendal nerve puncture from the dorsal direction from topographical and anatomical perspectives. They reported that the average insertion point of the needle was 14 cm in the greater trochanter of the femur, 9 cm above the ischial tuberosity, and 6 cm lateral to the gluteus. This enables the needle to have a large area of contact with the pudendal nerve, resulting in greater stimulation efficacy and lower stimulation intensity. Jottard et al. (2017) described a minimally invasive transbuttock endoscopic method for implanting pudendal electrodes for neuromodulation under full visual control. Konschake et al. (2017) accurately and reliably identified the pudendal nerve using laparoscopic techniques, and developed a direct, reliable, and minimally invasive pudendal neuroendoscopic surgical method relying on laparoscopic techniques.

The above-described methods are complex and require a precise, reversible, efficient, and minimally invasive method to puncture and localize the pudendal nerve. Three-dimensional printing technology is being used increasingly in modern medicine in various disciplines, including orthopedics (Lal and Patralekh, 2018) and maxillofacial surgery (Han et al., 2019), and Morrison et al. (2019) have developed a 3D printed device for cranial nerve stimulation in mice (Morrison et al., 2019). Yinjun et al. (2016) and Zhang et al. (2018), respectively, proposed the application of 3D printed technology in sacral neuromodulation, so as to reduce the difficulty of surgery and increase the effect of surgery. In the current study, 3D printed navigation was used to meet the aforementioned design requirements. Autopsy data were combined with pelvic and neurological patient data to achieve personalized procedural design. Neural modulation of the pudendal nerve was achieved by implanting the lead with the guidance of 3D printed navigation.

## Materials and Methods

### Acquisition of Underlying Data

**FIGURE 1:**
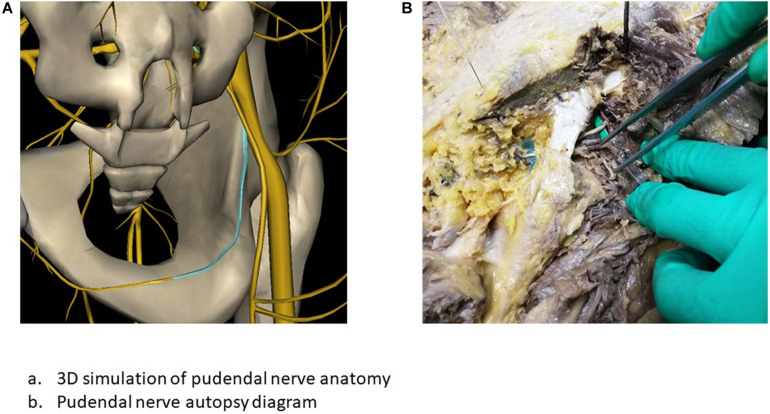
Anatomic localization of the pudendal nerve. **(A)** 3D simulation of pudendal nerve anatomy. **(B)** Pudendal nerve autopsy diagram.

The pudendal nerve is composed of nerve fibers arising in the S2–S4 nerve roots, and runs in the pudendal canal and accompanies the internal pudendal artery. From the sacral plexus, the pudendal nerve travels between the piriformis muscle and the coccygeus muscle, then passes through the greater sciatic foramen into the pelvis, spans the ischial spine, passes between the sacrospinous ligament and the sacrotuberous ligament, and terminates at the perineum. Inside the pudendal canal, which is formed by the fascia, the pudendal nerve usually gives rise to three branches: the inferior rectal nerve, the perineal nerve, and the dorsal penile/clitoral nerve (Pirro et al., 2009; Figure 1).

**FIGURE 2:**
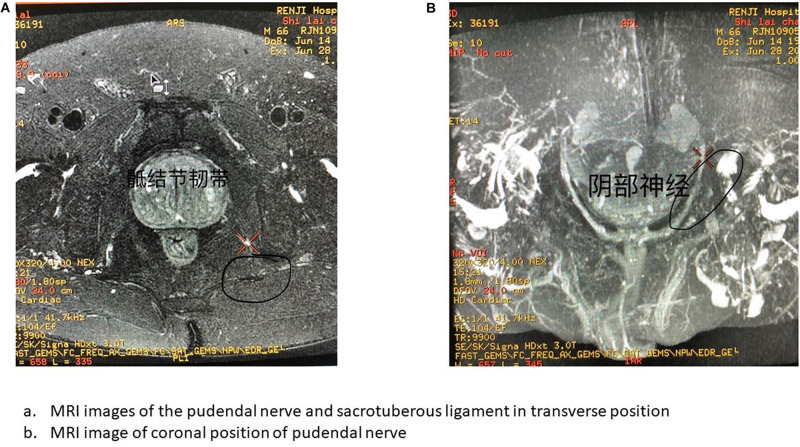
MRI image of pudendal nerve. **(A)** MRI images of the pudendal nerve and sacrotuberous ligament in transverse position. **(B)** MRI image of coronal position of pudendal nerve.

Two days before surgery, a four-point fixed positioning marker was placed on the patient’s body surface on the dorsal side of the sacrum, and marker points were marked with gentian violet to prevent bias in guide template placement. A plain computed tomography scan of the sacrum was conducted first using a UCT S-160 scanner (United Imaging, Shanghai, China). The scanning parameters used were tube potential 120 kV, tube current 200 mA, matrix, 512 × 512, field of view 150 mm, slice thickness 1.5 mm, spacing 1 mm, and pitch 0.9375, and 3D bone reconstruction was performed after scanning via a bone reconstruction algorithm. Magnetic resonance imaging (MRI) of the sacral and pudendal nerves was then conducted with a Signa HDXT 3.0T MRI scanner (General Electric, United States), using an 8-channel cardiac coil with the patient in the dorsal position and the foot advanced. Neuroimaging was performed using a 2D, thin-section, cross-sectional, fast-spin echo, fat suppression sequence (Axial T2 WI-FSE iDEAL). The scanning parameters were field of view 320 mm, matrix 320 × 224, repetition time 5380 ms, echo time 68 ms, echo train length 16, bandwidth 50 Hz, slice thickness 2 mm, spacing 0 mm, and number of excitations 1. The scanning range for continuous acquisition was from the lower edge of the L5 vertebral body to the lower edge of the S5 vertebral body (Figure 2).

### Navigation Template Design and 3D Printed

**FIGURE 3:**
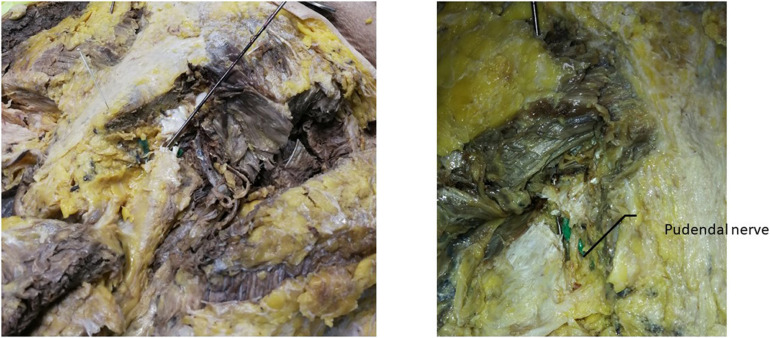
Ideal site for a postmortem puncture.

**FIGURE 4:**
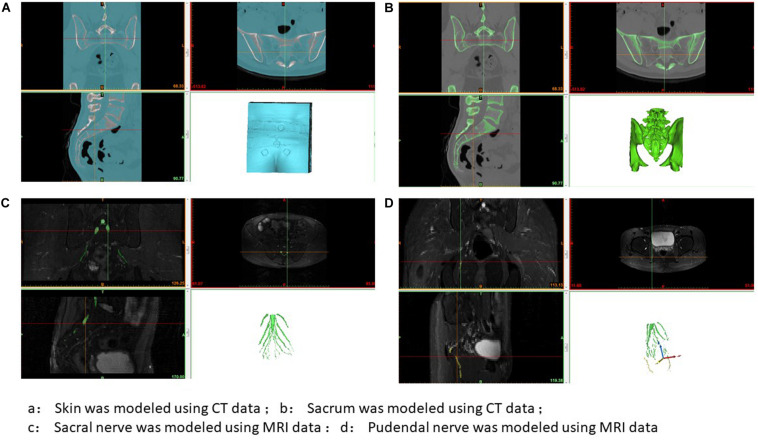
3D printing guide plate data extraction. **(A)** Skin was modeled using CT data; **(B)** Sacrum was modeled using CT data; **(C)** Sacral nerve was modeled using MRI data; **(D)** Pudendal nerve was modeled using MRI data.

**FIGURE 5:**
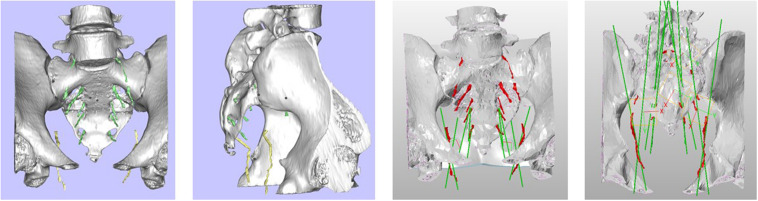
3D printing guide plate CT and MRI data fusion modeling.

**FIGURE 6:**
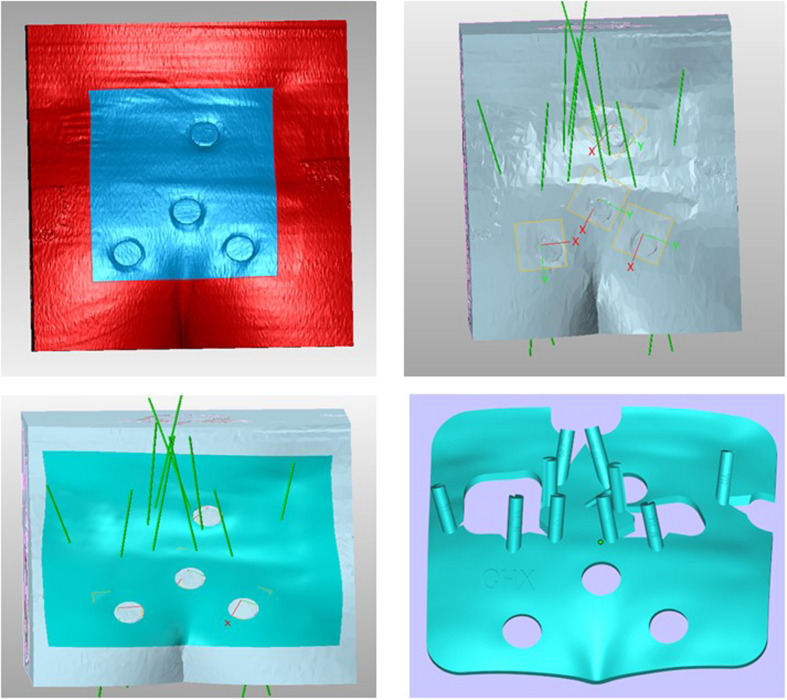
Puncture design and modeling of 3D printing guide plate.

Two methods were used to locate the pudendal nerve, autopsy studies and patient-based procedures. Autopsy studies were used to identify the correct position for the needle to ensure the largest contact area with the pudendal nerve. Puncture of the ischial tuberosity on the same side resulted in a larger contact area between the needle and the pudendal nerve (Figure 3). Based on puncture navigation derived from the individual patient’s data, the patient’s unique pelvic floor anatomy was obtained by fusing sacrum computed tomography imaging and pelvic floor nerve MRI, and the puncture needle path was designed with the requirement of maximum contact area between the puncture needle and the pudendal nerve (Figures 4–6). In either method, the location of the pudendal nerve should be combined with the patient’s own imaging data. So 3D printed navigation templates are personalized designs.

### Procedure

**FIGURE 7:**
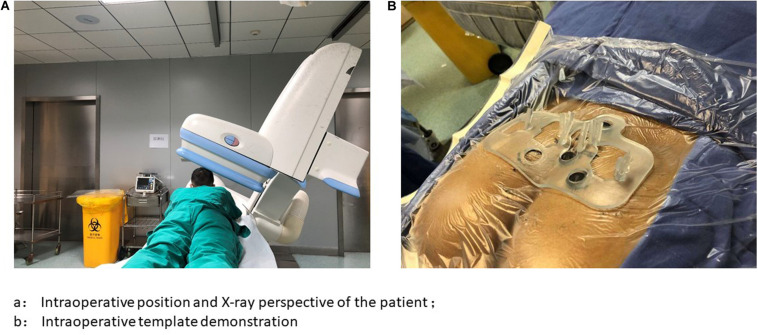
Presentation of X-ray perspective and surgical template. **(A)** Intraoperative position and X-ray perspective of the patient; **(B)** Intraoperative template demonstration.

**FIGURE 8:**
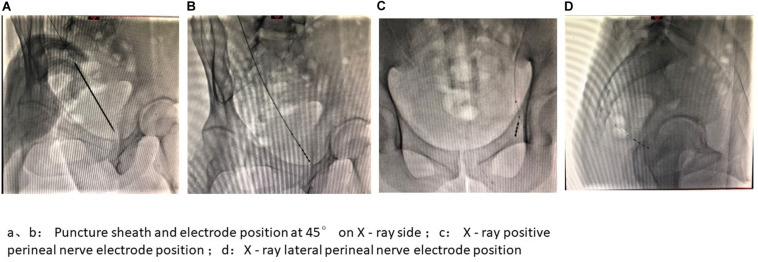
Intraoperative image presentation of PNM I. **(A,B)** Puncture sheath and electrode position at 45° on X-ray side; **(C)** X-ray positive perineal nerve electrode position; **(D)** X-ray lateral perineal nerve electrode position.

A standardized sacral nerve puncture procedure (Expert Consensus Writing Group on Clinical Application of Sacral Neuromodulation, 2017) was used throughout the entire study. The procedure was conducted as follows: (1) Place the patient in a prone position on a digital subtraction machine tool, with the leg slightly elevated and the knee joint bent to ensure toe suspension. (2) Disinfect and drape the sacrococcyx and hip surgical fields and expose the anal region and feet. (3) Match the 3D printed navigation template with the preoperative positioning markers, and perform local anesthesia with reference to the template puncture point. (4) Perform the puncture in accordance with the 3D printed navigation template, and observe the distance between the puncture needle and the ischial tuberosity 45° below the digital subtraction machine and adjust it. (5) When the needle position is satisfactory, test the patient’s motor and sensory responses to determine whether the access site is correct. (6) After confirming that the position of the needle is correct, remove the needle, insert the depth indicator needle, place the electrode introducer sheath containing the dilator along the depth indicator needle, and observe the position of the sheath 45° on the x-ray side, so that a “breakthrough feeling” is appropriate. (7) Remove the introducer sheath and depth indicator needle, leaving only the dilator. (8) Implant the self-fixing electrode. Once the test is satisfied, keep the barbed electrode still, remove the sheath, and fix the electrode. (9) Make a 3-cm incision in the outer upper right buttock for the permanent stimulator to be implanted into. Use a subcutaneous tunneler to draw out the barbed electrode tail end, connect the percutaneous extension, and guide the percutaneous extension out of the skin of the opposite buttock through the subcutaneous tunnel (10). Test each shock response and close the buttock incision (Figures 7, 8). The protocol was approved by the Shanghai Jiao Tong University School of Medicine.

During the operation, the sacral nerve electrode and pudendal nerve electrode were placed simultaneously. The degree of symptom improvement determined whether a permanent stimulator was placed and the location of the implanted nerve was determined. A Medtronic 3889 nerve stimulation electrode was used as the nerve stimulation electrode, a Medtronic 3531 Verify stimulator was used for phase I testing, and a Medtronic 3058 nerve stimulator was used as the phase II implantable stimulator, with parameter settings of frequency 2–45 Hz, pulse width 150–360 μs, and voltage of <3 v.

### Statistical Method

SPSS 20.0 software was used for statistical analysis. Measurement data are expressed as mean ± standard deviation. *T* test was used for pairwise comparison of measurement data, and *p* < 0.05 was considered statistically significant.

## Results

**FIGURE 9:**
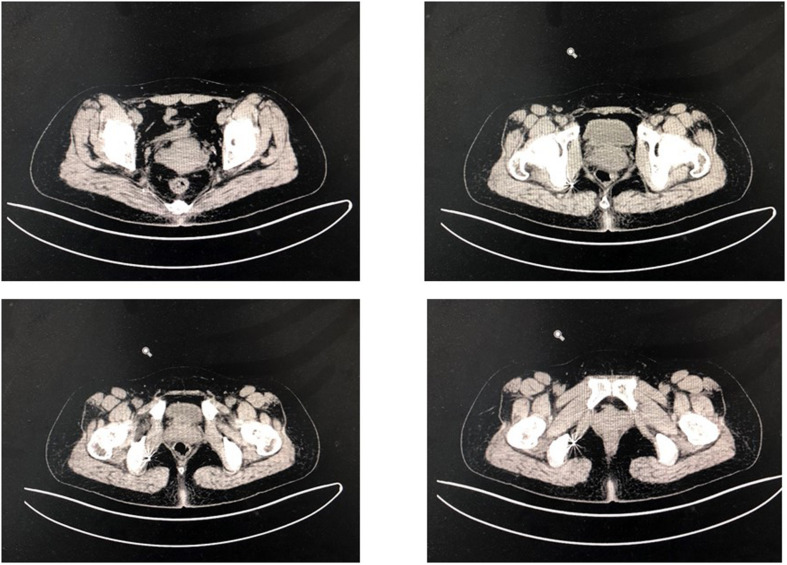
Imaging position of electrode 1 month after operation.

A total of 16 patients were included in the study. All patients who underwent surgery underwent postoperative pelvic floor computed tomography to assess whether the electrode position was adapted to the pudendal nerve and sacral nerve (Figure 9). Improvement of pain, urinary frequency, and dysuria were scored on a 0–10-point scale, where 0 represented no improvement and 10 represented complete improvement. Of a total of 16 patients, seven were male and nine were female, and their mean age was 50.56 years. There were seven patients with perineal pain, three patients with frequent urination and urgent urination, and six patients with poor urination. Five patients did not have a permanent stimulator, two patients were implanted with the sacral nerve, and nine patients were implanted with the perineal nerve.

The improvement of pain in the 16 patients (6.33 ± 1.63 points) was superior to the improvement in urinary frequency (3.71 ± 1.95 points) and dysuria (4.25 ± 1.71 points) (*p* < 0.05), but pudendal nerve stimulation also had an effect on urinary frequency and dysuria. No patients had any serious complications throughout the surgical treatment. Symptom scores are shown in Table 1.

**TABLE 1:** The clinical situation.


Serial number	Gender	Age	Main symptoms	Transformation situation	Dysuria improves symptoms	Improved urinary frequency	Pain relief*
1	F	77	Pain	NON	–	–	2.0
2	F	54	Pain	Pudendal nerve	–	3.0	6.0
3	M	25	Frequent urination	Pudendal nerve	–	4.0	–
4	F	77	Pain	Pudendal nerve	–	–	8.0
5	M	50	Dysuria	NON	2.0	–	–
6	M	33	Dysuria	Pudendal nerve	6.0	3.0	–
7	F	65	Pain	Pudendal nerve	–	–	7.0
8	F	80	Pain	Sacral nerve	–	5.0	5.0
9	M	41	Dysuria	Pudendal nerve	4.0	–	–
10	F	40	Frequent urination	Sacral nerve	–	5.0	4.0
11	M	30	Dysuria	Pudendal nerve	5.0	3.0	–
12	F	35	Pain	NON	–	–	3.0
13	F	44	Pain	Pudendal nerve	–	–	8.0
14	F	62	Dysuria	NON	0.0	–	–
15	M	44	Frequent urination	Pudendal nerve	–	3.0	6.0
16	M	52	Dysuria	NON	3.0	–	–

*The improvement of pain, urinary frequency, and dysuria were statistically different (*P* = 0.05 and *P* = 0.037).

## Discussion

Although electrical nerve stimulation is an effective treatment for neurological diseases, researchers continue to seek new neural targets to utilize in new methods in the field of neuroscience. Armstrong and Vancaillie (2016) previously described the case of a 35-year-old woman whose pain was greatly improved by sacral and pudendal nerve therapy. In a retrospective analysis, Peters et al. (2015) also reported that significant improvements in patients with pudendal neuralgia were achieved via chronic pudendal neuromodulation. These data are consistent with the preliminary data reported herein. As well as being effective for the treatment of pain, pudendal neuromodulation can also improve symptoms such as frequent urination (Wang et al., 2016, 2017). Notably, however, the pudendal nerve has no bony localization, so surgically locating it and puncturing it are problematic. For this reason the use of 3D printed technology to assist localization and puncture of the pudendal nerve was investigated in the present study. It is still the first time to be applied in the field of nerve regulation, especially in the pelvic floor and peripheral nerve.

3D printed navigation has the following advantages: (1) It is a personalized navigation based on each individual patient’s pelvic data, thus the surgery is more accurate and the electrode can better adapt to the pudendal nerve, resulting in better efficacy. (2) The surgical method draws on the traditional sacral nerve regulation surgical method, which is minimally invasive and reversible because it does not destroy surrounding tissues and nerves. (3) It is simple and convenient, and the surgeon can adapt to it by mastering the traditional sacral nerve regulation surgical method. (4) The patient does not require general anesthesia at any point during the procedure (local anesthesia is sufficient), and postoperative recovery is rapid and without serious complications.

3D printed navigation has some limitations. MRI pudendal nerve data are difficult to obtain. Different scanning parameters are required for different brands of instruments, and in the current study 8 h of MRI monitoring were required to obtain the MRI scanning parameters required to perform the procedure. Lastly, 3D printed navigation is not monitored, which renders the operation susceptible to error effects.

## Conclusion

In summary, pudendal nerve stimulation is a new therapeutic option for pelvic floor diseases. It can have beneficial effects on pain, frequent urination, and poor urination, but it may be most effective for treating pain. Utilizing a combination of autopsy data and patient-derived pelvic and nerve data, a personalized design method was developed in the current study. Neural modulation of the pudendal nerve was achieved by implanting a lead with the guidance of 3D printed navigation. Three-dimensional printing navigation can maximize the phase II conversion rate, reduce the difficulty of surgery, shorten the operation time, reduce damage to additional organs and blood vessels, and increase the accuracy of implanted electrodes, and it can be performed while the patient is awake. This surgical method is accurate, reversible, efficient, and minimally invasive.

## Data Availability Statement

The original contributions presented in the study are included in the article/supplementary material, further inquiries can be directed to the corresponding author.

## Ethics Statement

The studies involving human participants were reviewed and approved by the Renji Hospital, Shanghai Jiao Tong University School of Medicine. The patients/participants provided their written informed consent to participate in this study. Written informed consent was obtained from the individual(s) for the publication of any potentially identifiable images or data included in this article.

## Author Contributions

YG: data curation and original manuscript draft preparation. TL: software operation and data validation. CJ: patients pay a return visit. JL: supervision and manuscript writing, reviewing, and editing. All authors contributed to the article and approved the submitted version.

## Conflict of Interest

The authors declare that the research was conducted in the absence of any commercial or financial relationships that could be construed as a potential conflict of interest.
